# Behavioural and Genetic Evidence for *C. elegans*' Ability to Detect Volatile Chemicals Associated with Explosives

**DOI:** 10.1371/journal.pone.0012615

**Published:** 2010-09-07

**Authors:** Chunyan Liao, Andrew Gock, Michelle Michie, Bethany Morton, Alisha Anderson, Stephen Trowell

**Affiliations:** CSIRO Entomology and CSIRO Food Futures Flagship, Canberra, Australian Capital Territory, Australia; Brown University, United States of America

## Abstract

**Background:**

Automated standoff detection and classification of explosives based on their characteristic vapours would be highly desirable. Biologically derived odorant receptors have potential as the explosive recognition element in novel biosensors. *Caenorhabditis elegans*' genome contains over 1,000 uncharacterised candidate chemosensory receptors. It was not known whether any of these respond to volatile chemicals derived from or associated with explosives.

**Methodology/Principal Findings:**

We assayed *C. elegans* for chemotactic responses to chemical vapours of explosives and compounds associated with explosives. *C. elegans* failed to respond to many of the explosive materials themselves but showed strong chemotaxis with a number of compounds associated with commercial or homemade explosives. Genetic mutant strains were used to identify the likely neuronal location of a putative receptor responding to cyclohexanone, which is a contaminant of some compounded explosives, and to identify the specific transduction pathway involved. Upper limits on the sensitivity of the nematode were calculated. A sensory adaptation protocol was used to estimate the receptive range of the receptor.

**Conclusions/Significance:**

The results suggest that *C. elegans* may be a convenient source of highly sensitive, narrowly tuned receptors to detect a range of explosive-associated volatiles.

## Introduction

Automated standoff detection and classification of explosives based on their characteristic vapours would be highly desirable. Although some homemade explosives and taggants have higher vapour pressures [1], commercial and military explosives have vapour pressures at ambient temperature ranging from 10^−6^ to 10^−14^ molecules per molecule of air [2]. This makes detecting such explosives extremely challenging. Canines can detect some compounds down to 10^−15^ g mL^−1^ [1], equivalent to 10^–13^ molecules per molecule of air for a compound with M ≈200. There is also evidence that dogs may learn to detect explosives by virtue of signature compounds other than the energetic compounds themselves [3], [4], [5]. Such signature compounds may include solvents and precursors involved in the manufacture and formulation of explosives, as well as breakdown products and taggants, many of which have relatively high vapour pressures. Canines therefore offer an excellent combination of sensitivity, discrimination and adaptability for explosive sniffing and are still used widely for this purpose [3], [4], [5].

Instrument-based biosensing is an alternative to canines that is being investigated for automated vapour detection of explosives [5], [6]. So far, most biosensor-based approaches to explosive detection have used antibodies as recognition elements. However, the ongoing use of dogs, reports that insects can be trained to detect explosive vapours [7], [8] and electrophysiological data obtained from insects [9] indicate that biologically-derived odorant receptors have potential as explosive sensors. Furthermore, the limits of detection for a few odorant receptor sensitivities fall in the picomolar range for selected compounds in the aqueous phase [10], [11], [12], equivalent to 10^−13^–10^−14^ molecules of odorant per molecule of water. This has led to efforts to identify biological odorant receptors that are specific for explosive signature compounds. Radhika *et al*. [13] previously identified a rat receptor that responds to 2,4-dinitrotoluene, a component of a number of explosives.

Nematodes, like insects and mammals, have a well-developed chemosensory system. *Caenorhabditis elegans* is known to detect more than 100 volatile compounds from many chemical classes including alcohols, ketones, esters, aldehydes and aromatics [14], [15]. Its genome contains over 1,000 uncharacterised candidate chemosensory receptors [16], [17], [18], which belong to the same G-protein coupled receptor (GPCR) superfamily as mammalian odorant receptors. It is therefore possible that *C. elegans* would be a source of sensors for detection of explosive-associated volatiles. However, unlike mammals or insects, we are unaware of any evidence that *C. elegans* can detect or respond to chemicals that constitute volatile signatures for explosives. To build a sensor array for any diverse set of chemicals, it is not necessary to match a sensor to every chemical. Such an approach would be impractical. However it is a requirement that the sensors as a group adequately cover the relevant odorant space defined by the set of chemicals of interest. We therefore screened *C. elegans* for chemotactic responses to chemical vapours relevant to a range of explosives and obtained several hits. We show that the nematode responds to some chemicals known to occur in the headspace of commercial or homemade explosives. Genetic mutant strains were used to identify the likely neuronal location of a putative receptor responding to cyclohexanone and to identify the specific transduction pathway involved. Upper limits on the sensitivity of the nematode were calculated. A sensory adaptation protocol was used to estimate the receptive range of the receptor. The results suggest that *C. elegans* may be a convenient source of highly sensitive, narrowly tuned receptors for a range of explosive-associated volatiles.

## Results and Discussion

Using a population chemotaxis assay, wild-type *C. elegans* were screened for responses to 17 chemicals associated with explosives, including high explosives and solvents, precursors, breakdown products and other potential contaminants of commercial and home made explosives (Supplementary Table S1). These chemicals were selected from nine different chemical classes. Ten compounds (acetone, 2-butanone, nitromethane, cyclohexanone, hydrogen peroxide, potassium perchlorate, RDX, hexamine, sulphur and potassium nitrate) when diluted 1/1000, stimulated chemotactic responses that were significantly different (p<0.05) from the ethanol response based on T-tests (Table 1). The hydrogen peroxide response was still significant when tested at 1/100 but the hexamine response was not. In addition, when tested at 1/100 nitroglycerine, also gave a highly statistically significant response. We must interpret these results with caution for several reasons. Firstly, there is a high level of variability in chemotaxis data, including the ethanol control. Secondly, given the number of compounds we tested there is a high probability that at least one statistically significant result could occur by chance. Finally, the assay is conducted as a choice test, in which chemotaxis to the odorant diluted in ethanol, a known attractant [15], competes with chemotaxis to ethanol alone. On the other hand ANOVA, which only identified the responses to nitroglycerine (1/100) and RDX, hexamine and potassium nitrate (1/1000) as significant (Table 1), appears to be too conservative, as other information, including tests at different concentrations, confirms the biological relevance of several of the positive assays. In this case, the results of a significance test should only be used as a preliminary indication that a nematode responds to a particular chemical. In view of the positive CI for the ethanol control, consistent with [15], we would be particularly cautious about placing too much weight on low negative chemotaxis indices such as those generated by TNT, potassium chlorate, RDX and hexamine, unless supported by other evidence.

**Table 1:** Results of screening *C. elegans* for chemotaxis to a range of odorants associated with home-made and commercial explosives.

Compound	Dilution	Mean CI	SEM	t Test (P)	Class	Category	ANOVA
Nitroglycerine	1/100	0.83	0.05	<0.001	nitroalkane	explosive	*
Acetone	1/1000	0.79	0.07	0.001	ketone	precursor	
2-Butanone	1/1000	0.79	0.08	0.001	ketone	precursor	
Nitromethane	1/1000	0.50	0.07	0.027	nitroalkane	precursor	
Nitroglycerine	1/1000	0.47	0.23	0.182	nitroalkane	explosive	
Hexamine	1/100	0.47	0.08	0.085	heterocycle	precursor	
Cyclohexanone	1/1000	0.46	0.09	0.040	ketone	contaminant	
RDX	1/100	0.27	0.12	0.810	nitroamine	explosive	
Ethanol (control)	1/1000	0.23	0.11	n/a	–	–	
TATP	1/100	0.09	0.14	0.443	peroxide	explosive	
TATP	1/1000	0.06	0.11	0.143	peroxide	explosive	
Potassium chlorate	1/1000	0.05	0.27	0.290	inorganic salt	precursor	
PETN	1/100	0.05	0.08	0.214	nitroalkane	explosive	
Ethyl hexanol	1/100	0.05	0.23	0.370	alcohol	contaminant	
TNT	1/1000	0.04	0.17	0.180	nitroaromatic	explosive	
PETN	1/1000	0.04	0.16	0.183	nitroalkane	explosive	
Dimethyldinitrobutane	1/100	0.02	0.15	0.303	other	explosive	
Dimethyldinitrobutane	1/1000	−0.11	0.27	0.150	other	explosive	
Hydrogen peroxide	1/1000	−0.14	0.16	0.048	peroxide	precursor	
Potassium perchlorate	1/1000	−0.19	0.04	0.004	inorganic salt	precursor	
Ethyl hexanol	1/1000	−0.22	0.23	0.070	alcohol	contaminant	
TNT	1/100	−0.23	0.15	0.049	nitroaromatic	explosive	
Potassium chlorate	1/100	−0.25	0.07	0.002	inorganic salt	precursor	
Hydrogen peroxide	1/100	−0.25	0.12	0.012	peroxide	precursor	
RDX	1/1000	−0.26	0.11	0.007	nitro amine	explosive	*
Hexamine	1/1000	−0.28	0.12	0.008	heterocycle	precursor	*
Sulphur	1/1000	−0.32	0.09	0.002	element	precursor	
Potassium nitrate	1/1000	−0.41	0.21	0.018	inorganic salt	precursor	*

CI - Chemotaxis index of the mean of at least four biological repeats of two plates each (except potassium perchlorate with two repeats and the ethanol control with one biological repeat comprising one plate) as defined in Materials and Methods, SEM – Standard error of mean. t Test shows P value relative to the ethanol control. ANOVA: * indicates p<0.05 in a one way ANOVA and Dunnett's post test comparing all means to the ethanol control. Note that ethanol itself is a known chemoattractant for *C. elegans* [15].

TATP - Triacetone triperoxide; TNT – Trinitrotoluene; PEPN - Pentaerythritol tetranitrate; RDX – Cyclotrimethylenetrinitramine.

Soluble compounds were generally screened as 1 µL droplets of a 1/1000 dilution. For volatile compounds, the highest concentrations initially detected by the nematodes in the vapour phase are of the order of 3 parts per million by volume (ppmv) or less (for calculation see below). Of the chemicals we tested because of their association with explosives, only the responses of acetone and 2-butanone have previously been reported. The hit rate we observed was similar to that reported by Bargmann *et al.* [15] indicating that *C. elegans* may express receptors that respond to a significant proportion of volatiles selected for reasons unconnected with and not obviously relevant to nematode biology. This response repertoire goes beyond what seems likely to be used as food cues and may reflect a broader ability of the nematode to sense its environment including detection of predators, environmental toxins or pathogens. Lack of a chemotactic response to one of the test compounds does not necessarily indicate the absence of a relevant receptor but could be due to the lack of a chemotactic response or a balance, at the concentration tested, between positive and negative chemotactic responses. On the other hand, a reliable chemotactic response is a clear indication that the worm expresses at least one receptor that binds the compound tested.

The response to cyclohexanone was selected for further investigation for the following reasons. Firstly, cyclohexanone is known to be present in some explosive formulations and has been shown to be the most abundant constituent of the headspace over C-4 plastic explosive [3]. Secondly, it has been shown that some trained explosive sniffer dogs may use cyclohexanone as a detection cue [3], [5]. Finally, the response to cyclohexanone, CI = 0.46±0.09 with p = 0.04, was of intermediate strength and statistical significance. A successful strategy for characterising the basis of the cyclohexanone response would therefore likely be more broadly applicable.

**Figure 1:**
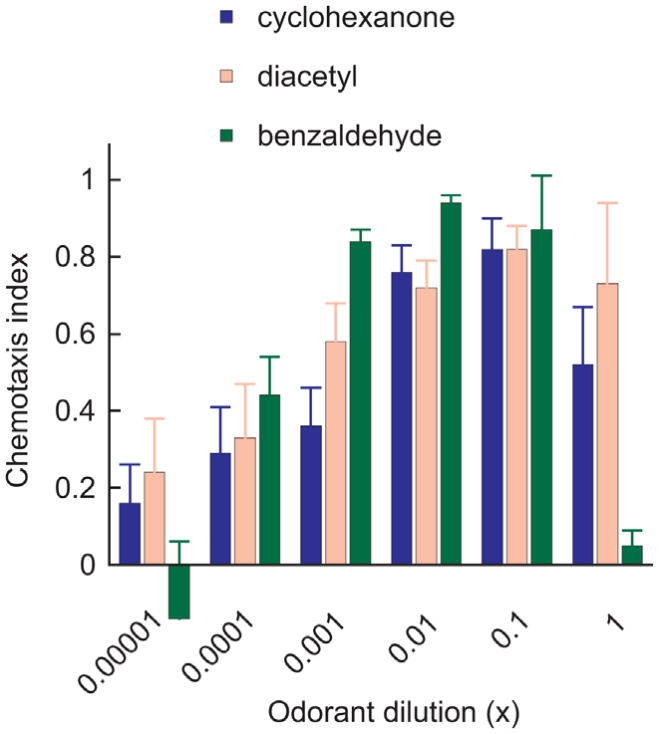
*C. elegans* chemotaxis indicates the presence of a high affinity receptor for cyclohexanone. Log concentration-response curves for the chemotaxis of wild-type N2 nematodes to cyclohexanone (blue) compared with the responses to the AWA-directed odorant diacetyl (pink) and AWC-directed odorant benzaldehyde (green). Each bar represents the mean ± sem of at least four independent assays involving two plates each.

Adult *C. elegans* worms were attracted strongly to cyclohexanone over a broad range of concentrations (Figure 1). However, despite the obvious trend, the response was only significantly different from control down to a dilution of a 1/1,000. Assuming complete evaporation and uniform distribution of cyclohexanone in the 80 mL headspace above the test agar, one microlitre of a 1/1,000 dilution of cyclohexanone corresponds to a mean concentration of approximately 3 parts per million by volume (ppmv) in air. Assuming ideal gas behaviour, this equates to 3×10^−6^ molecules of odorant per molecule of air. This is an upper limit for the sensitivity of receptors that underlie the cyclohexanone behavioural response. Diffusion, convection and mixing with external air will tend to decrease the concentration available to the nematode, implying a limit of detection at the receptor that is probably below ppmv. Furthermore, in our hands, the nematodes' behavioural sensitivity to cyclohexanone is broadly similar to their sensitivity to diacetyl or benzaldehyde (Figure 1) and the sensitivity of the isolated ODR-10 receptor to diacetyl is below parts per trillion by volume (pers. comm. Helen Dacres). In the presence of 1 µl of undiluted cyclohexanone, nematodes showed a marked reduction in response relative to that seen at the one in ten dilution. Such repellancy at higher concentrations is commonly seen with otherwise attractive odorants.

### Cellular origin of the cyclohexanone response

**Figure 2:**
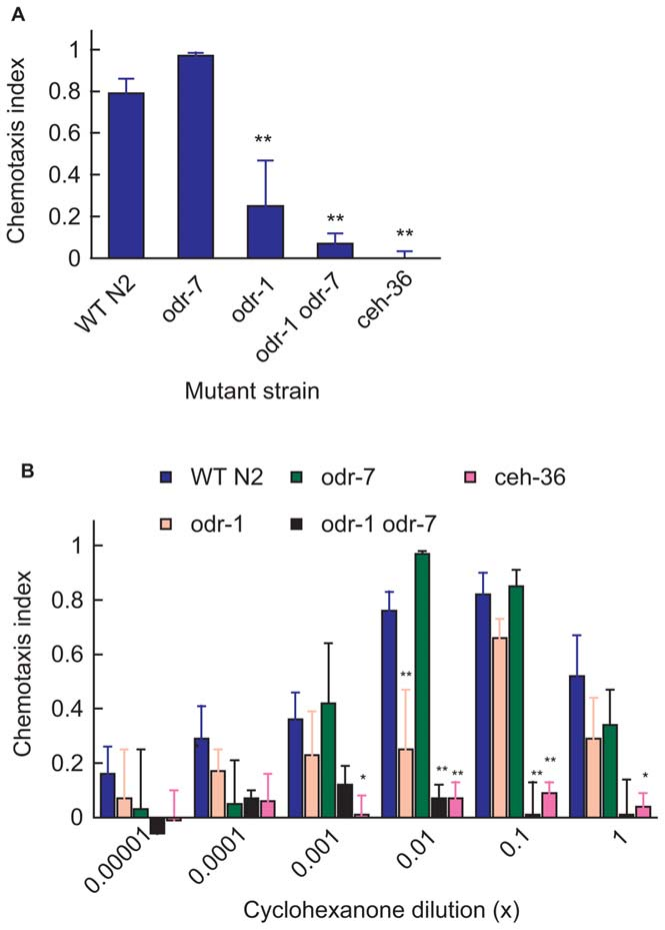
The *C. elegans* chemotactic response to cyclohexanone is primarily mediated via the AWC neurones. (A) Chemotactic responses of wild-type N2 and cell specific mutants *odr-7(ky4), odr-1(n1936), odr-1*(*n1936) odr-7(ky4)* and *ceh-36(ky646)* to 1∶100 cyclohexanone. (B) Log-concentration chemotactic responses of wild-type and cell specific mutants to cyclohexanone. N2 (blue), *odr-1(n1936)* (pink), *odr-7(ky4)* (green), *odr-1(n1936) odr-7(ky4)* (pink line), and *ceh-36(ky646)* (purple line). Each bar represents the mean ± sem of at least six (A) or four (B) independent assays. Statistics: * p<0.05 and ** p<0.01 in a one way ANOVA and Dunnett's post test comparing all means to the wild-type (N2) mean.

The primary chemosensory organs in *C. elegans* are known as amphids, a pair of organs located either side of the mouth. Each amphid contains 12 chemosensory neuron types. Positive chemotactic responses to volatile odorants have long been known to originate from the AWA/AWC neuron pairs [15]. Repellant responses originate predominantly in the AWB pair of neurons [18]. Because the predominant response to cyclohexanone vapour is positive chemotaxis, we assumed that at least one cyclohexanone-responsive receptor is expressed in the AWA and/or the AWC neurons. To test this assumption, we exploited mutant lines of *C. elegans* with known defects in cell specification or cell-specific components of the olfactory transduction pathway. We used *odr-7*, *ceh-36* and *odr-1* chemotaxis mutants. *odr-7(ky4)* mutants fail to respond to any of the odorants normally detected by AWA neurons but their AWC function is intact [19]. It is believed that in *odr-7* mutants the AWA neurons are reprogrammed to resemble AWC and mis-express a subset of AWC markers [20]. The *odr-7(ky4)* strain exhibited a slightly stronger attraction to cyclohexanone than wild type at a dilution of 1∶100 (Figure 2A) although at higher or lower concentrations, the response was similar to or substantially weaker than wild type (Figure 2B). *ceh-36* is an otx-like homeobox gene which specifies the identity of AWC olfactory neurones. *ceh-36* null mutants fail to respond to any AWC-sensed odorants [21], [22], [23]. The deletion and likely null mutation, *ceh-36(ky646)* [21] abolished attraction to cyclohexanone over a wide range of concentrations (Figure 2). The allele *ceh-36(ks86)*, which is a missense mutation with a severe effect [24], was tested at a 1/100 dilution and also failed to elicit a response (not shown). To eliminate possible involvement of ASE neurons in the loss of responsiveness by *ceh-36* mutants, we also tested a *che-1* line, which is defective in the specification of ASE [25]. At a 10^–2^ dilution of cyclohexanone, the chemotaxis index for *che-1* was 0.76±0.10, indicating that abolition of cyclohexanone chemotaxis by *ceh-36* mutants is due to defects in AWC rather than ASE.

Because odr-7 mutants are, if anything, enhanced in their responses to cyclohexanone and ceh-36 mutants have lost cyclohexanone responsiveness, we conclude that AWC is the predominant cellular origin of cyclohexanone responsiveness. AWA appears to make a lesser contribution. To confirm the role of AWC neurons in the detection of cyclohexanone, we tested the responses of the *odr-1(n1936)* mutant. The ODR-1 protein, a guanylyl cyclase involved in downstream odorant signalling, is expressed in AWC, AWB, ASI, ASJ and ASK but not AWA neurons. *odr-1* mutants are therefore defective in AWC and AWB-mediated olfaction [26]. Responses to cyclohexanone were reduced in the mutant compared with wild type over a range concentrations (Figure 2) and this difference was statistically significantly at the 1/100 dilution (**p<0.01 for 10^−2^ cyclohexanone). However *odr-1(n1936)* retained a positive response at lower dilutions, which contrasts with its negative response for some other odorants [26] and we do not have a complete explanation for this behaviour. We also generated an *odr-7(ky4) odr-1(n1936)* double mutant, which is expected to lack both AWA and AWC neurons and is not attracted to diacetyl nor to benzaldehyde (data not shown). Relative to the *odr-7* single mutation, the double mutant exhibited a significantly lower response to cyclohexanone down to 1/1000 dilution (Figure 2A and B). The responses of the *odr 1 odr7* were also significantly lower than the *odr 1* line at higher concentrations (p = 0.0002 at 1/10; p = 0.48 at 1/1) possibly indicating the involvement of AWA at higher concentrations of cyclohexanone. However, the lack of response from the *ceh-36(ky646)* mutant over a broad range of dilutions strongly implicates a high affinity cyclohexanone receptor that is normally expressed in AWC neurones.

### Transductional coupling of the putative cyclohexanone receptor

It is useful to know the transduction pathways involved in cyclohexanone sensing, in order to identify proteins that might directly contact the putative cyclohexanone receptor. Olfactory receptors in *C. elegans* are G-protein coupled receptors and follow the general scheme of G-protein mediated transduction. In the AWA neuron, odorant receptors activate the G-proteins ODR-3 and/or GPA-3 [27], [28]. This results in the activation of a TRP-V-like ion channel, comprising OSM-9 and other subunits. Although phospholipase C is involved in other transduction systems that incorporate TRP-channels, the precise mechanism of channel activation remains unknown [29]. A third G-protein, GPA-5, seems to have a lesser, inhibitory, role [28]. In the AWC neuron, odorant receptors also signal through ODR-3 and GPA-3 G_θ_ subunits [27], [28] but a number of other G-proteins are involved in modulating transduction. The AWC transduction cascade also involves a guanylate cyclase [26], [30], [31] and a cyclic-nucleotide-gated ion channel [32]. A recent study suggests that the AWB olfactory transduction pathway closely resembles that of AWC [33].

To identify the cyclohexanone receptor's interactions with G proteins and other elements of the GPCR transduction cascade, we therefore conducted chemotaxis assays using mutants defective for the relevant G_α_ subunits, guanylyl cyclases and ion channels.

**Figure 3:**
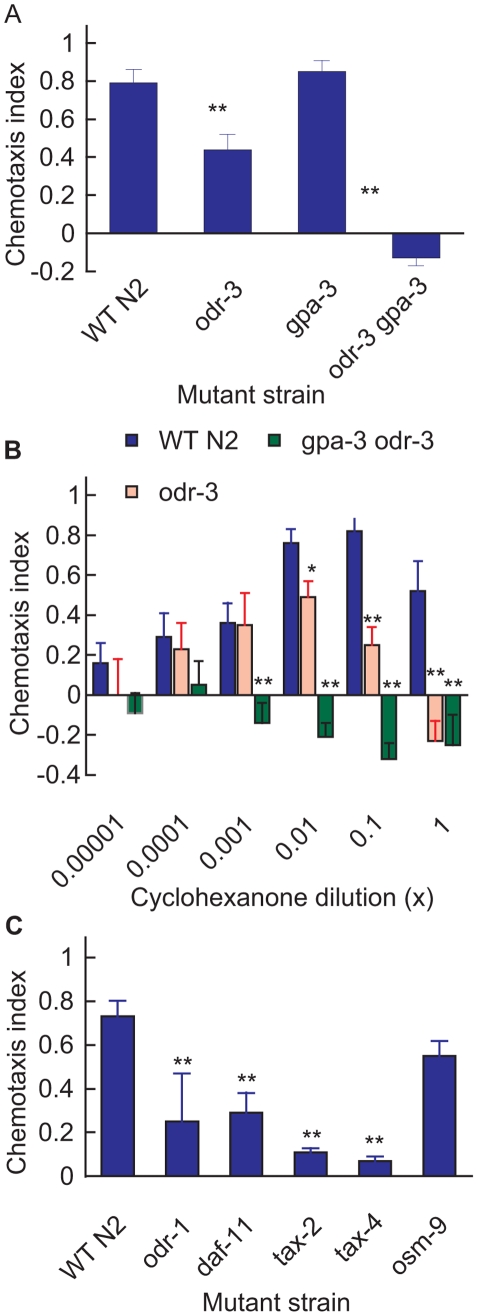
Genetic analysis of cyclohexanone receptor transduction pathway. (A) Chemotactic responses of wild-type N2 and mutants defective in one or more G_α_ subunits: *odr-3(n2150), gpa-3(pk35),* and *gpa-3(pk35) odr-3(n1605)* to 1∶100 cyclohexanone. (B) Log cyclohexanone concentration-response data for wild type N2 (blue), mutant *odr-3(n2150)* (pink) and *gpa-3(pk35) odr-3(n1605)* (green). (C) Chemotactic responses of mutant lines defective in various guanylate cyclase or ion-channel subunits: *odr-1(n1936), daf-11(m47), tax-2(ks10), tax-4(ks28),* and *osm-9(ok1677)* to 1∶100 cyclohexanone. Each bar represents the mean ± sem of at least six (A, C) or four (B) independent assays. Statistics: * p<0.05 and ** p<0.01 in a one way ANOVA and Dunnett's post test comparing all means to the wild-type (N2) mean.

Chemotaxis towards 1/100 cyclohexanone was depressed approximately 40% in the *odr-3(n2150)* mutant (**p<0.01; Figure 3A). *odr-3* is a nonsense mutation, which is defective in chemotaxis to all odorants sensed by the AWA or AWC neurons [27]. Chemotaxis was also reduced or reversed at higher concentrations (Figure 3B). However there was no significant change in chemotaxis index in the *gpa-3(pk35)* mutant line, which is a null allele [34], [35], nor in lines carrying mutations in the GPA-2, GPA-2 & GPA-3, GPA-13, GPA-5 G_α_ proteins (results not shown). It is known that GPA-3 can substitute for ODR-3 in the latter's absence [28]. We therefore tested NL2105 *odr-3(n1605) gpa-3(pk35)*, a line carrying mutations in both the *odr-3* and *gpa-3* genes. The latter double mutant failed to respond to any odorant normally sensed by either the AWA or AWC neurons, demonstrating that the stimulatory role of GPA-3 in nematode olfaction is redundant to ODR-3 [28]. As predicted, cyclohexanone chemotaxis was completely abolished in the double mutant (Figure 3). These data indicate that a receptor for cyclohexanone signals predominantly through the *odr-3* and/or *gpa-3* pathways, i.e. it conforms to the general model for AWC transduction as described by Lans *et al.* [28].

Generally speaking, all mutants with defects downstream of the G-protein in the signal transduction pathway of AWC cells showed severely defective chemotactic responses to 1∶100 cyclohexanone (Figure 3C). The ODR-1 and DAF-11 are subunits of an AWC guanylate cyclase and are believed to function as a heterodimer [30]. As mentioned previously, *odr-1(n1936)* mutants showed depressed chemotactic responses to 1∶100 cyclohexanone. Similarly *daf-11(m47)* mutants responded only 40% as well as the N2 strain (Figure 3C). The residual chemotactic responses to cyclohexanone of *odr-1(n1936)* and *daf-11(m47)* imply that either subunit may partially complement the loss of the other.

The cyclic nucleotide gated cation channel TAX-2/TAX-4, which is essential for many sensory processes, is expressed in AWC and other sensory neurons but not AWA [31], [32], [36]. Mutations in either one of the channel subunits, *tax-2(ks10)* and *tax-4(ks28)*, severely depressed nematode responses to cyclohexanone. In contrast, the chemotactic responses of nematodes carrying the AWA signal transduction channel mutation *osm-9(ok1677)*, which is required for all known functions of the AWA olfactory neurons [37], [38], [39], were not statistically different from responses of the wild-type strain (Figure 3C). We interpret the non-significant decrease in the *osm-9(ok1677)* strain's chemotaxis, relative to N2, to the former's generally poor vigour and movement rather than any specific olfactory defect.

### Chemical selectivity of the putative cyclohexanone receptor of *C. elegans*

Without an isolated receptor there is no direct way to measure the responses of a particular nematode receptor to a range of chemicals. Accordingly, to investigate the odorant-specificity of the putative cyclohexanone receptor, we used exposure to low levels of cyclohexanone to drive adaptation of the putative receptor's response, followed by challenge with a test odorant. When the response to a challenge odorant is ablated by adaptation with cyclohexanone, it is not proven that the challenge odorant binds and stimulates the same receptor. Adaptation could be occurring downstream of the receptor in the transduction pathway. However, where exposure to low levels of cyclohexanone does not adapt the response to a second chemical it clearly indicates that the latter does not stimulate the putative cyclohexanone receptor.

**Figure 4:**
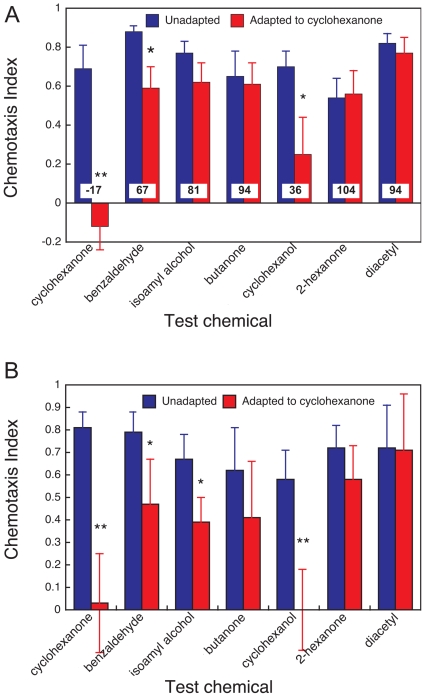
Cyclohexanone receptor selectivity probed by odour adaptation. Wild-type N2 worms were adapted to 1 µl of 1/10 (A) or undiluted (B) cyclohexanone for 60 minutes prior to chemotaxis to the specified odorants being measured. Control worms were treated identically except that odorant was not present during the adaptation period. Dilutions of test odorants were: cyclohexanone 1∶100, benzaldehyde 1∶100, isoamyl alcohol 1∶100, cyclohexanol 10 mg/ml, butanone 1∶1000, diacetyl 1∶1000. Bars represent the mean ± sem of at least six independent assays. Statistics: * p<0.05 and ** p<0.01 comparing the mean of adapted to the mean of unadapted nematodes. Numbers (A) indicate the adapted response as a percentage of the control, unadapted response to the test odorant.

Using the adaptation protocol described in Materials and Methods, we investigated the effect of adaptation on responses to a range of chemicals that share some chemical functionality or molecular structure with cyclohexanone. We were unable to obtain consistent responses (dose-dependent responses) below an adapting dose of 1 µL of a 1/10 dilution of cyclohexanone. The volume of adapting stimulus used here was either 3–5 or 30–50 fold lower compared with studies investigating adaptation with other odorants such as diacetyl [40], or benzaldehyde [41]. Nevertheless, robust odorant-specific adaptation was observed. Worms adapted with 1 µL of 1/10 or undiluted cyclohexanone completely lost responsiveness to cyclohexanone at 1∶100 (Figure 4). As expected, there was no cross-adaption to 1/1000 diacetyl, which is sensed by AWA, at either adapting dose. At the lower adapting dose (Figure 4A), butanone, 2-hexanone and isoamyl alcohol were not cross-adapted by cyclohexanone. Of the test chemicals, only the responses to cyclohexanol (10 mg mL^−1^) and benzaldehyde (10^−2^) were substantially adapted, dropping to 36–67% of the control chemotaxis index. At the higher adapting dose of 1 µL undiluted cyclohexanone, greater levels of adaptation were observed and only the response to diacetyl was completely unaffected (Figure 4B). The responses to 2-butanone and 2-hexanone were slightly reduced but differences between the adapted and unadapted groups were not statistically significant. Pre-incubation with cyclohexanone suppressed the response to cyclohexanol as effectively as it did the response cyclohexanone itself (**p<0.01). Benzaldehyde and isoamyl alcohol were also substantially adapted (*p<0.05 vs mean of unadapted worm).

These data imply that the putative cyclohexanone receptor shows very tight odorant tuning because a discriminating adapting dose (0.1 µL cyclohexanone) was found where none of the responses to test chemicals were adapted to the same extent as the cyclohexanone response.

*C. elegans* adaptation is likely caused by inactivation of receptors or other elements of the transduction cascade [41]. Vertebrate and Drosophila GPCRs are regulated by kinases of the ßARK/rhodopsin kinase family and arrestins [42]. The kinases phosphorylate and down-regulate ligand-bound receptors, a process known to initiate light adaptation of rhodopsin responsiveness [43], [44]. In the nematode, adaptation involves the uncharacterised *adp-1* gene, the TRPV channel subunit OSM-9, and the cGMP-dependent protein kinase EGL-4 [41], [45]. Since odorant adaptation is triggered by calcium and cGMP levels, it has been suggested that adaptation is regulated through G-protein subunits [28].

Partial rather than complete adaptation could be explained by cross-talk between chemicals at the receptor level. For example, partial adaptation would be seen if cyclohexanol is a weaker ligand for the receptor than cyclohexanone or if cyclohexanone adaptation partially reduces the responsiveness of a cyclohexanol receptor. A second possible explanation is based on the concept that adaptation can propagate through the transduction tree, from the receptors (twigs) through the G-proteins (branches) to the cyclase and ion-channels (main trunk). The stronger an adapting stimulus, the more likely it is that adaptation will occur at lower levels in the tree, temporarily blocking responses to all higher-level stimuli and receptors.

Odorant sensation and adaptation are distinct processes. For example, *adp-1* and *osm-9* mutants are defective in adaptation to different subsets of AWC-sensed odorants but neither mutation alters the unadapted chemotactic response to AWC-sensed odorants [41]. It has been suggested that adaptation may influence odorant preferences over intermediate time-scales (minutes to hours) allowing the animal to select among odorants based on its recent experience [41].

Field detection and identification of a range of volatile chemicals, including explosives, is particularly challenging when they are present at low levels and/or in the presence of variable interfering backgrounds. Effective chemical sensor arrays minimally comprise an array of semi-broad and overlapping sensors, which must be independent of each other in odorant space [46]. However, the performance of such an array is likely to be enhanced by supplementation with high affinity, highly selective receptors targeted at volatiles of particular interest. Insect pheromone receptors have exactly these characteristics of high affinity and selectivity. A number of insect general odorant receptors also respond to very low odorant concentrations and a small proportion of them are narrowly tuned [46], [47].

It is estimated that the nematode *C. elegans* may have more than 1000 chemoreceptors [16], [17]. The worm is capable of detecting more than a hundred odorants representing many chemical classes [15], [48], [49]). The number of receptors is of the same order of magnitude as the number of candidate receptors in some mammalian species and more than are expressed in any insect species. Behavioural experiments indicate the worm shows parts per billion, or better, sensitivity to a number of odorants. However, the small number of neurons in the nematode's nervous system cannot support complex combinatorial coding of odours. It has therefore been suggested that nematode olfactory receptors will be found to be highly specific with, perhaps, only one or two receptor types responding per odorant at physiological odorant concentrations [50]. The only nematode odorant receptor to be characterised to date seems to conform to this pattern. The ODR-10 receptor, when expressed in mammalian cells, did not respond to the volatile odorants 2,3-pentanedione and butanone, which are minimally different from the known ligand, 2,3-butanedione [51]. The likely high selectivity of nematode olfactory receptors in combination with their undoubted sensitivity makes them good candidates for detecting key odorants under field conditions. In this study we demonstrate, for the first time, that nematodes express receptors that respond to a significant region of odorant space relevant to explosive detection. We selected a putative cyclohexanone receptor to demonstrate the feasibility of using *C. elegans* mutant lines to define a receptor's cellular and molecular characteristics. Practical exploitation of *C. elegans* receptors for explosive detection will require development of a transduction system to make odorant detection machine-readable and, minimally, targeted “de-orphaning” of relevant nematode chemoreceptors.

### Conclusions

Here we have demonstrated that *C. elegans* responds behaviourally to a number of volatiles derived from or associated with explosives. We identified a putative high affinity receptor for cyclohexanone, a solvent used in explosive formulation that is a prevalent headspace volatile over C-4 explosive. Although the statistically validated limit of detection for cyclohexanone in the behavioural assay was 3 ppmv, experience suggests that receptors underlying this type of response are likely to have a much lower limit of detection. The putative receptor is predominantly expressed in the AWC neuron and signals through the G_α_ subunit ODR-3. Signal transduction also involves the guanylate cyclase subunits ODR-1 and DAF-11 and the cyclic nucleotide gated channel subunits TAX-2 and TAX-4. A limited set of adaptation experiments indicate that the putative receptor is narrowly tuned. The results provide motivation and a rationale for isolating the cyclohexanone receptor and other receptors that respond to explosive associated compounds. Localisation of the putative cyclohexanone receptor to the AWC neuron and identification of proteins, such as ODR-3 with which the receptor interacts, will facilitate efforts to isolate and characterise it. For example, armed with this knowledge it would now be possible to narrow the search for a cyclohexanone receptor by functional expression of a subset of chemoreceptor transcripts expressed in the AWC neuron and/or by using the ODR-3 G_α_ as a probe to identify interacting GPCRs.

## Materials and Methods

### Strains and genetics

All nematodes were grown on Petri plates with *E. coli* strain OP50 at 21°C under standard conditions [52]. The following *C. elegans* strains were provided by the *Caenorhabditis elegans* Genetics Centre at The University of Minnesota: Bristol N2, mutant strains CX4 *odr-7(ky4)* X, FK311 *ceh-36(ks86)* X, CX5893 *kyls140* I;*ceh-36(ky646)* X, CX2065 *odr-1(n1936)* X, DR47 *daf-11(m47)* V, CX2205 *odr-3(n2150)* V, NL334 *gpa-2(pk16)* V, NL335 *gpa-3(pk35)* V, NL348 *gpa-2(pk16) V gpa-3*(pk35) V, NL1137 *gpa-5(pk376)* X, NL2330 *gpa-13(pk1270)* V, VC1262 *osm-9(ok1677)* IV, FK100 *tax-2(ks10)*I, FK103 *tax-4(ks28)* III, PR679 *che-1(p679)* I. The double mutant: NL2105 *odr-3(n1605)* V *gpa-3(pk35)* V was provided by Dr. Gert Jansen of The Erasmus Medical Centre, Rotterdam. The double mutant strain: TZ0018 *odr-1 (n1936)* X *odr-7(ky4)* X was generated in our laboratory by standard methods. The identities of double mutant strains were confirmed by PCR and, where necessary, by sequencing.

### Chemotaxis assays

Well-fed young adult and adult worms were used in the assay. Chemotaxis assays were performed essentially as described by Bargmann *et al.* [15]. The assay was conducted on 90 mm diameter ×20 mm deep Petri plates. Glass lids were used to minimise odorant absorbance. Odorant in solvent (1 µL) and pure solvent (1 µL) were applied on opposite sides of the glass lids except that 5 µL was used where the solvent was water. Milli-Q water was used to wash worms off culture plates. Adult worms were separated from early instars, bacteria and other potential attractants by filtration through a 20 µm nylon mesh. Ten minutes before placing worms onto the assay plates, 1 µL droplets of 0.5 M sodium azide were applied to the surface of the agar at the location of the attractant and the negative control to immobilise worms reaching either position. Chemotaxis assays were performed with ≥100 worms at room temperature for 60 minutes. At the end of the assay, the glass lid was removed and the plate was inverted over a filter paper impregnated with 0.3 mL chloroform for 1 minute to immobilise the worms, which were counted under a dissecting microscope. Negative control assays were performed in which a pure 1 µL ethanol target was provided at each end of the plate.

Odorants were obtained from Sigma or provided by Dr. Paul Kirkbride of the Australian Federal Police. TATP was provided by Dr. Mark Fitzgerald of Weapons Systems Division, Defence Science and Technology Organisation, Australia. Odorants were diluted in ethanol (AllTech, HPLC grade) except potassium nitrate, potassium perchlorate and potassium peroxide, which were diluted in water (AllTech, HLPC grate).

For screening odorants associated with home-made and commercial explosives, a standard 1∶1000 dilution was used. Where p values in the original screen were >0.05, additional tests were performed using a dilution of 1∶100. With two exceptions noted in Table 1, tests were conducted in duplicate and a minimum of four tests was performed on two or more different occasions, i.e. eight plates in total. For assays using mutant strains, cyclohexanone was diluted 1∶100 in ethanol. Each test was conducted on duplicate plates and at least six tests were conducted for each odorant, with tests conducted on two or more different days. For cyclohexanone concentration-response assays, serial ten-fold dilutions (from undiluted to 1×10^−5^) were prepared and at least four tests were performed. All odorant dilutions were made within 24 hours of the assay.

### Cylohexanone adaptation assay

Cyclohexanone adaptation assays were performed essentially as described by [41]. Three plates of well-fed worms were washed with Milli-Q water over a 20 µm nylon mesh. Retained worms were placed on the surface of an agar plate and excess water was removed with filter paper. Cyclohexanone, neat or diluted in HPLC grade water, was distributed among five agar plugs on the glass lid of the plate. The plate was sealed with parafilm and incubated for 60 minutes at room temperature. After adaptation, assay buffer (5 mM Phosphate, pH 6, 1 mM CaCl_2_ and 1 mM MgSO_4_) was used to wash worms into a 15 mL conical centrifuge tube. Assay buffer was used to bring the final volume to 10 ml and the worm suspension was allowed to settle for 5 minutes. The supernatant was removed and the suspension of worms was placed in a well of a 96-well plate prior to being used in the chemotaxis assay as described.

Control unadapted worms were treated identically, except that no odorant was added to the agar plugs. Each plate, containing approximately 100 worms, was assayed for chemotaxis as described above. Adapted and non-adapted worms were tested for responses to 1∶100 cyclohexanone, 1∶100 benzaldehyde, 1∶100 isoamyl alcohol, 1∶1000 2-butanone, 10 mg/ml (w/v) cyclohexanol, 1∶10 2-hexanone, 1∶1000 diacetyl for 60 minutes. At least six repeats (n = 6–8) were performed for each odorant.

### Statistical analysis

The chemotaxis index (CI) was calculated as the number of worms in the odorant zone minus the number of worms in the control zone, divided by the total number of worms [15]. Means represent data pooled from assays run on at least two different days with four to eight repeats. Error bars in all figures are standard error of means. The data obtained were analysed using a two-tailed T-test and one-way ANOVA with Dunnett's post test using GraphPad InStat version 3.00 for Windows 95 or GraphPad Prism v 5.0c for Mac (GraphPad Software, San Diego California USA).

## Supporting Information

Table S1 Relevance of tested compounds to home-made, commercial and military explosives. This information is provided for convenience and is compiled from a number of publically available sources, including: Oxley JC, Smith JL, Shinde K, Moran J (2005) Determination of the vapor density of triacetone triperoxide (TATP) using a gas chromatography headspace technique. Propellants Explosives Pyrotechnics 30: 127–130, Material Safety Data Sheets and the relevant Wikipedia pages. TATP - Triacetone triperoxide; TNT - Trinitrotoluene; PETN - Pentaerythritol tetranitrate; RDX - Cyclotrimethylenetrinitramine.
(0.05 MB DOC)
