# Supplementary material for: Behavioural and Genetic Evidence for C. elegans' Ability to Detect Volatile Chemicals Associated with Explosives
Source: PLoS One. 2010 Sep 7;5(9):e12615. doi: 10.1371/journal.pone.0012615 (PMC2935383; doi:10.1371/journal.pone.0012615)
Supplement: Table S1 — Relevance of tested compounds to home-made, commercial and military explosives. This information is provided for convenience and is compiled from a number of publically available sources, including: Oxley JC, Smith JL, Shinde K, Moran J (2005) Determination of the vapor density of triacetone triperoxide (TATP) using a gas chromatography headspace technique. Propellants Explosives Pyrotechnics 30: 127–130, Material Safety Data Sheets and the relevant Wikipedia pages. TATP - Triacetone triperoxide; TNT - Trinitrotoluene; PETN - Pentaerythritol tetranitrate; RDX - Cyclotrimethylenetrinitramine. (0.05 MB DOC) [file pone.0012615.s001.doc]

**Supplementary Table 1. Relevance of tested compounds to home-made, commercial and military explosives**

| **Compound** | **Molecular formula** | **Molecular weight** | **Vapour pressure ρ (kPa)** | **Temperature (^o^C) ρ measured** | **Relevance to explosives** |
| --- | --- | --- | --- | --- | --- |
| Acetone | C_3_H_6_O | 58.08 | 24 | 20 | Solvent used in manufacture |
| 2-Butanone | C_4_H_8_O | 72.11 | 9.47 | 20 | Solvent used in manufacture |
| Cyclohexanone | C_6_H_10_O | 98.16 | 0.67 | 20 | A solvent used in the manufacture of C-4 |
| Dimethyldinitrobutane | C_6_H_12_N_2_O_4_ | 176.17 | 2.79 x 10^-4^ | 25 | ICAO taggant for plastic explosives |
| Ethyl hexanol | C_8_H_18_O | 130.23 | 0.6 | 20 | A solvent used in the manufacture of C-4 |
| Hexamine | C_6_H_12_N_4_ | 140.18 | negligible |  | A solid fuel and component of detonators for high explosives |
| Hydrogen peroxide | H_2_O_2_ | 34.02 | 0.67 | 30 | A component of home made explosives |
| Nitroglycerine | C_3_H_5_N_3_O_9_ | 227.09 | 3.47 x 10^-5^ | 20 | A component of industrial explosives |
| Nitromethane | CH_3_NO_2_ | 61.04 | 3.7 | 20 | A component of an explosive mixture known as ANNM |
| PETN | C_5_H_8_N_4_O_12_ | 316.14 | 5 x 10^-11^ | 25 | Military high explosive |
| Potassium chlorate | KClO_3_ | 123 | negligible |  | Oxidizing agent. Used to manufacture explosives and matches |
| Potassium nitrate | KNO_3_ | 101.11 | negligible |  | An explosive primer, an oxidiser in black powder |
| Potassium perchlorate | KClO_4_ | 138.55 | negligible |  | An oxidizer used in ammunition percussion caps, explosive primers, propellants, flash compositions, stars, and sparklers |
| RDX | C_3_H_6_N_6_O_6_ | 222.12 | 1.86 x 10^-10^ | 25 | Military high explosive |
| Sulphur | S_8_ | 32.07 | 0.133 | 184 | Component of gunpowder |
| TATP | C_6_H_12_O_4_ | 222.24 | 7 x 10^-3^ | 25 | Home made explosive |
| TNT | C_7_H_5_N_3_O_6_ | 227.3 | 5 x 10^-7^ | 25 | Explosive |

This information is provided for convenience and is compiled from a number of publically available sources, including: Oxley JC, Smith JL, Shinde K, Moran J (2005) Determination of the vapor density of triacetone triperoxide (TATP) using a gas chromatography headspace technique. Propellants Explosives Pyrotechnics 30: 127-130, Material Safety Data Sheets and the relevant Wikipedia pages. TATP - Triacetone triperoxide; TNT – Trinitrotoluene; PETN - Pentaerythritol tetranitrate; RDX – Cyclotrimethylenetrinitramine.
